# Natural Mutations in *Streptococcus agalactiae* Resulting in Abrogation of β Antigen Production

**DOI:** 10.1371/journal.pone.0128426

**Published:** 2015-06-05

**Authors:** Anastasia Vasilyeva, Ilda Santos Sanches, Carlos Florindo, Alexander Dmitriev

**Affiliations:** 1 Department of Molecular Microbiology, Institute of Experimental Medicine, Saint-Petersburg, Russia; 2 Department of Life Sciences, Centro de Recursos Microbiológicos (CREM) and Research Unit on Applied Molecular Biosciences (UCIBIO, REQUIMTE), Faculdade de Ciências e Tecnologia, Universidade Nova de Lisboa, Caparica, Portugal; 3 Department of Infectious Diseases, National Institute of Health, Lisbon, Portugal; 4 Department of Fundamental Problems of Medicine and Medical Technologies, Saint-Petersburg State University, Saint-Petersburg, Russia; University of Kansas Medical Center, UNITED STATES

## Abstract

*Streptococcus agalactiae* genome encodes 21 two-component systems (TCS) and a variety of regulatory proteins in order to control gene expression. One of the TCS, BgrRS, comprising the BgrR DNA-binding regulatory protein and BgrS sensor histidine kinase, was discovered within a putative virulence island. BgrRS influences cell metabolism and positively control the expression of *bac* gene, coding for β antigen at transcriptional level. Inactivation of *bgrR* abrogated *bac* gene expression and increased virulence properties of *S*. *agalactiae*. In this study, a total of 140 strains were screened for the presence of *bac* gene, and the TCS *bgrR* and *bgrS* genes. A total of 53 strains carried the *bac*, *bgrR* and *bgrS* genes. Most of them (48 strains) expressed β antigen, while five strains did not express β antigen. Three strains, in which *bac* gene sequence was intact, while *bgrR* and/or *bgrS* genes had mutations, and expression of β antigen was absent, were complemented with a constructed plasmid pBgrRS(P) encoding functionally active *bgrR* and *bgrS* gene alleles. This procedure restored expression of β antigen indicating the crucial regulatory role of TCS BgrRS. The complemented strain A49V/BgrRS demonstrated attenuated virulence in intraperitoneal mice model of *S*. *agalactiae* infection compared to parental strain A49V. In conclusion we showed that disruption of β antigen expression is associated with: i) insertion of ISS*a4* upstream the *bac* gene just after the ribosomal binding site; ii) point mutation G342A resulting a stop codon TGA within the *bac* gene and a truncated form of β antigen; iii) single deletion (G) in position 439 of the *bgrR* gene resulting in a frameshift and the loss of DNA-binding domain of the BgrR protein, and iv) single base substitutions in *bgrR* and *bgrS* genes causing single amino acid substitutions in BgrR (Arg187Lys) and BgrS (Arg252Gln). The fact that BgrRS negatively controls virulent properties of *S*. *agalactiae* gives a novel clue for understanding of *S*. *agalactiae* adaptation to the human.

## Introduction

*Streptococcus agalactiae* (group B streptococcus) is a gram-positive bacterium which is able to cause the broad spectrum of human and animal diseases such as pathologies of the pregnancy, invasive infections of newborns (sepsis, meningitis, pneumonia), abscesses, endocarditis, mastitis of the dairy cows and other infections of different tissues and organs [1, 2, 3]. In order to successfully escape from the pressure of the host immune system and effectively colonize numerous tissues, this bacterium employs variety of the virulence factors which expression is coordinated at transcriptional, translational and post-translational levels. In particular, the expression of *S*. *agalactiae* virulence factors is controlled at transcriptional level by two-component regulatory systems (TCSs) and global transcriptional regulators [4].

The major function of the TCSs is sensing environmental changes and further modulation of the changes in expression of different proteins. TCSs consist of two proteins. The first protein, histidine kinase, senses the environmental changes and autophosphorylates a conserved histidine residue, and then transfers this phosphoryl group to the second protein, DNA-binding response regulator. This reaction results in conformational changes in DNA-binding response regulator molecule providing an ability to function as transcriptional regulator (activator or repressor) by binding with gene promoters through DNA binding domain.

At present, a total of 21 TCSs were identified in *S*. *agalactiae* [5–16]. They were found to be important for the control of *S*. *agalactiae* metabolism and expression of virulence factors. For example, DNA-binding response regulator CovR positively controls transcription of CAMP factor *cfb* gene and negatively controls transcription of hemolysin *cylE* gene and C5a peptidase *scpB* gene [7, 8]. At phenotypic level the *covR* mutant strain was characterized by attenuated virulent properties compared to the parental wild-type strain [8]. Microarray analysis identified the CovRS core-regulon. It included aminopeptidase C, serine peptidase, membrane protein, CAMP factor, oxidoreductase, hemolysin genes, and variety of hypothetical genes, among others [9]. *S*. *agalactiae* two-component system DltR/DltS is important for transcription of *dltABCD* operon, which is necessary for synthesis of lipoteichoic acids. Inactivation of *dltA* and *dltR* genes resulted in the attenuated virulent properties of *S*. *agalactiae in vivo* [10]. RgfA/RgfC TCS controls adhesion of *S*. *agalactiae* to epithelial cells and affects transcription of *scpB* gene [11]. FspSR TCS-16 was suggested to be involved in bacterial fitness and carbon metabolism during host colonization [16].

BgrRS two-component system of *S*. *agalactiae* was found to be located within the putative virulence island of 8992 bp in size [12], and BgrR and BgrS proteins shared 83% and 78% similarity with *S*. *pneumoniae* RR06 DNA-binding response regulator and HK06 histidine kinase, respectively [17]. It is suggested that this island was recently acquired by *S*. *agalactiae*, and an acquisition of this island provided certain selective advantages to *S*. *agalactiae* [18, 19]. The co-transcribed genes of BgrRS, *bgrR* and *bgrS*, are adjacent to the virulence gene *bac* encoding for the surface β antigen [12]. This surface protein has capacity to bind IgA and factor H of complement, and it is considered to be an important virulence factor [18–22].

Given the significance of *bac* gene (or β antigen) for *S*. *agalactiae*, the current studies are aimed to investigate the occurrence of *bac* gene (or β antigen) among *S*. *agalactiae* strains of different serotypes, among *S*. *agalactiae* strains of both human and animal origins, and among invasive and non-invasive strains [23–25]. The *bac* gene is also suggested to be used as one of genetic marker for microarray-based typing scheme of *S*. *agalactiae* isolates [26]. Certain studies are currently performed in order to evaluate the functional role of β antigen as vaccine candidate component [27, 28] and analyze its role in pathogenesis of *S*. *agalactiae* diseases [29].

Recently we constructed *bgrR* mutant and *bgrR+bgrS* double mutant *S*. *agalactiae* strains, and studied the functional role of BgrRS two-component system [14]. It was demonstrated that both transcription of *bac* gene and expression of encoded β-antigen were controlled by BgrR response regulator, but not BgrS histidine kinase. It was also found that regulation occurred at transcriptional level. In addition, inactivation of *bgrR* gene, but not *bgrS* gene, significantly affected virulence of *S*. *agalactiae* [14]. Together, these data demonstrated the functional role of BgrRS two-component system in regulation of β antigen and virulent properties of *S*. *agalactiae*.

As mentioned above, β antigen is considered to be an important virulence factor of *S*. *agalactiae*. However, similarly to other virulence factors of pathogenic bacteria, the expression of β antigen can vary in different strains or even be abrogated [30]. The goal of the present study was to screen a collection of *S*. *agalactiae* strains in order to reveal the strains without β antigen expression, to discover the reasons of abrogated β antigen expression, and to further study functional role of BgrRS TCS and β antigen in *S*. *agalactiae* virulence.

## Materials and Methods

### Bacterial strains and growth conditions

A total of 140 *S*. *agalactiae* human strains from microbiological collections in China (31 strains; Beijing Children’s Hospital), Russia (14 strains; Institute of Experimental Medicine, Saint-Petersburg), Sweden (15 strains; Lund University) and Portugal (80 strains, Faculdade de Ciências e Tecnologia. Universidade Nova de Lisboa and Instituto Nacional de Saúde Dr. Ricardo Jorge) associated with different diseases were used in the study. The strains belonged to serological types Ia (33 strains), Ib (32 strains), II (37 strains), III (15 strains), V (21 strains) and NT (2 strains). Previously described *S*. *agalactiae* 168/00 strain [14] was used as control strain. *S*. *agalactiae* strains were cultured overnight at 37°C in Todd-Hewitt Broth (THB) (HiMedia Laboratories Pvt. Ltd., India) or on THB blood agar plates containing 5% of sheep erythrocytes. *E*. *coli* strain DH5α was grown in Luria-Bertani (LB) broth (Sigma, USA) or on 1% LB agar plates. For *S*. *agalactiae* the following antibiotics were used: erythromycin, 2.5 μg/ml; spectinomycin, 125 μg/ml. For *E*. *coli* the following antibiotics were used: erythromycin, 200 μg/ml; spectinomycin, 100 μg/ml; of ampicillin, 100 μg/ml.

### DNA techniques

Routine DNA techniques were done according to [31]. Chromosomal DNA was isolated with DNA Express Kit (Liteh, Russia). Plasmid DNA was purified using the AxyPrep Plasmid Midiprep Kit (Axygen Biosciences, USA). PCR was carried out as described [14] using the primers listed in the Table 1. PCR with primers BgrF and BgrR was carried out with initial denaturation of 2 min at 94°C followed by 10 cycles of first step amplification (40 sec at 94°C, 50 sec at 35°C, and 90 sec at 72°C) and 30 cycles of second step amplification (40 sec at 94°C, 50 sec at 55°C, and 90 sec at 72°C). PCR products were purified with AxyPrep DNA Gel Extraction Kit (Axygen Biosciences). Sequencing of PCR products was performed by GenomeLab GeXP Genetic Analysis System using the GenomeLab Dye Terminator Cycle Sequencing with Quick Start Kit (Beckman Coulter, USA) and the primers listed in Table 1. Transformation of *E*. *coli* and *S*. *agalactiae* strains with recombinant plasmid was performed by Gene Pulser Xcell (Bio-Rad Laboratories, USA) as recommended by the manufacturer.

**Table 1:** Primers used in the study.

Primer name	Nucleotide sequence (5’– 3’)	Gene/Plasmid
L5	TGTAAAGGACGATAGTGTGAAGAC	*bac*
L6	CATTTGTGATTCCCTTTTGC	*bac*
BAC1	CACTGATACTGGAAAACGAGAGAAA	*bac*
BAC2	TTTAGAATCTTTCTGCTCTGGTGTT	*bac*
BAC3	GCCAGAAACTCCAGATACACCGAAG	*bac*
BAC4	ATTGGAAGGGTATACTGTAGAT	*bac*
BAC5	CTACAGTTGACAAAGAGCCT	*bac*
0524–76	AGCTTATGCTTGTCAATAATCACA	*bac*
0407–172	CACAAATGGAGATGCTGACTAG	*bac*
0407–173	CTAGTCAGCATCTCCATTTGTG	*bac*
0408–150	CTGAGCTAGGTATTAAGTCGCAAC	IS*Sa4*
0216–106	TCCCTATACAGCTGATGGCAT	IS*Sa4*
BgrF	CGGGATCCCGGAGTCTAATATCTGA	*bgrR* (upstream)
BgrR	CCGGAATTCCGTTCTTTTACTTTAT	*bgrS* (downstream)
hkFor	GGTCATCCACATTGCCATCTATC	*bgrS*
hkRev	GTCAAAACCTTATCCAATGCCTT	*bgrS*
rrFor	GATATGATTAGAGAGGGAGTAGCTGCT	*bgrR*
rrRev	GACCTGGTTCTTATGTTGGATCAA	*bgrR*
40/1	AGGAGGGACAGCTGGATATTACG	pVA891-2
40/2	TCCCATTTAGCCGTCATTTCAG	pVA891-2

### Construction of the recombinant plasmid pBgrRS(P)

In order to construct the recombinant plasmid expressing two-component regulatory system BgrRS, the following strategy was used. The primers BgrF and BgrR containing *Bam*HI and *Eco*RI restriction sites, respectively, were used to amplify DNA fragment containing entire *bgrR* and *bgrS* genes of the strain 168/00. This PCR fragment was digested with *Bam*HI and *Eco*RI, and ligated with spectinomycin resistant vector pAT29 digested with the same enzymes. After transformation of *E*. *coli* strain DH5α, one of spectinomycin resistant clones was selected. Isolation and sequencing of the recombinant plasmid confirmed insertion of the *Bam*HI-*Eco*RI DNA fragment containing both *bgrR* and *bgrS* genes in pAT29. The recombinant plasmid of 8900 bp in size was named as pBgrRS(P) and used in the following experiments.

### SDS-PAGE and Western-blotting

*S*. *agalactiae* strains were grown for 13 hours, and the cells were harvested by centrifugation. Bacterial lysates were prepared by 10 min boiling of the cells in 10% solution of 2-mercaptoethanol. The lysates were analyzed by SDS-PAGE as described [32]. The gels were stained with Coomassee R-250 (Amresco, USA). For Western-blotting the gels were electroblotted onto nitrocellulose membranes, 0.45 μm, (Bio-Rad Laboratories) as previously described [32, 33]. The conjugated human IgA-horseradish peroxidase was used to detect β antigen expression.

### Ethics Statement

Outbred ten-week old male mice were obtained from Rappolovo Animal Facility, Russia. All the experiments were performed according to the Protocol No. 3 (2011) approved by Animal Care Unit Committee, Institute of Experimental Medicine, Russia. The animals were housed in polycarbonate cages. Free access to balanced food and water was provided. After the experiments, all animals were sacrificed by CO_2_ asphyxiation and cervical dislocation.

### Murine infection model

Modeling of streptococcal infection was accomplished as previously described [14, 34]. Briefly, *S*. *agalactiae* clinical strain A49V (II serotype, China) that does not express β antigen due to a single nucleotide deletion resulting in a frameshift mutation of *bgrR* gene and the complemented strain A49V/BgrRS with functionally active two-component regulatory system BgrRS were grown in 40 ml of THB. The supernatants were removed, and the cells were washed with PBS several times, and resuspended in 4 ml of PBS. Ten-week old (14–16 g), male, white outbread mice (Rappolovo Animal Facility, Russia) were used in the study. The model of intra-peritoneal infection was used (S1 Table.). To do so, the each animal was infected by 0.5 ml of PBS containing 10^8^ CFUs of the strain. A total of 13 animals were used in each experimental group. As control, injection of PBS (0.5 ml) was applied to one additional group of 10 animals. Observation for laboratory animals, definition of the endpoints for sacrifice, as well as euthanasia protocol were recently published [34]. Ten days after infection all survived mice were also sacrificed. The spleens of all animals were homogenized in PBS, and the ten-fold dilutions of suspensions were grown at THB blood agar plates in order to determine the number of *S*. *agalactiae* CFUs. The large number of bacterial CFUs was isolated from the spleens of died animals. They were found to be group B streptococcal gram-positive, catalase negative, and identified as *S*. *agalactiae*. At the same time, *S*. *agalactiae* was not isolated from the spleens of survived animals and animals of the control group.

### Statistical analysis

The virulence of the strains was analyzed by Kaplan-Meier survival curve and the log-rank test. In order to identify statistically significant data (P values less than 0.05), the GraphPad Prism software (GraphPad Software Inc.) was used.

### Nucleotide sequence accession numbers

Gene sequences analyzed in this study have been deposited into the GenBank database (KF444678, KF444679, KF472175, KF472176 and HQ840774).

## Results

### Screening of the collection of *S*. *agalactiae* strains

**Fig 1:**
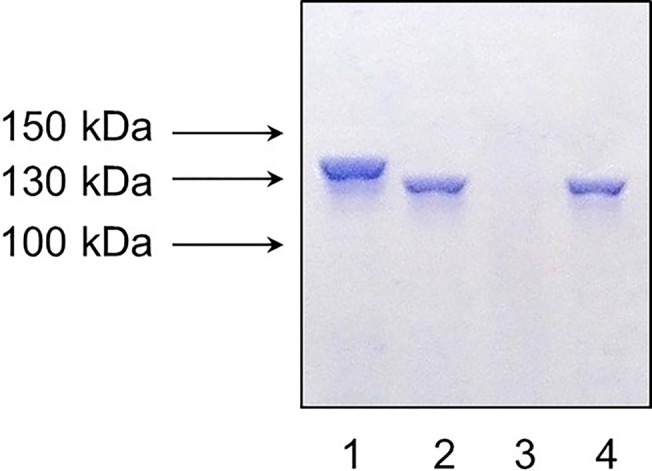
SDS-PAGE of *S*. *agalactiae* cell lysates followed by western-blotting with conjugated human IgA-horseradish peroxidase. Lanes 1, 2, 4: the strains that express β antigen; lane 3: strain that doesn’t express β antigen.

A total of 140 *S*. *agalactiae* strains were tested for the presence of *bac* gene by PCR using the primers L5 and L6. As result, 53 out of 140 strains were found to be *bac* gene positive: 16 out of 31 strains from China, 6 out of 14 strains from Russia, 7 out of 15 strains from Sweden and 24 out of 80 strains from Portugal (Table 2). Most of the *bac* gene positive strains represented Ib and II serotypes (Table 2). Additional PCR analysis of these 53 *bac* gene positive strains using the pairs of primers rrFor, rrRev and hkFor, hkRev demonstrated the presence of DNA-binding protein gene *bgrR* and sensor histidine kinase gene *bgrS*, respectively, in all the strains. Finally, all of these 53 *bac*, *bgrR* and *bgrS* gene positive strains were screened for the presence of β antigen by SDS-PAGE and western-blotting (Fig 1). A total of 48 strains expressed β antigen of different sizes which corresponds to the previous data indicating that in different strains the size of *bac* gene can be different as much as 0,5 kb [35]. However, 5 out of 53 *bac* gene positive strains did not produce β antigen: #8 (Ib serotype, Portugal), #06/08 (Ib serotype, Portugal), #122 (Ib serotype, Portugal), #128 (Ia serotype, Portugal), and A49V (II serotype, China). These strains were selected for further study.

**Table 2:** Amount of the *bac* gene positive strains of different serotypes.

	Ia	Ib	II	III	V	NT	
China	2	1	12	0	0	1	16 / 31
Russia	0	4	2	0	0	0	6 / 14
Sweden	0	5	1	0	0	1	7 / 15
Portugal	1	17	6	0	0	0	24 / 80
Total	53 / 140 (37.9%)						

### Insertion of the IS*Sa4* results in abrogation of β antigen expression in the strain #8

**Fig 2:**
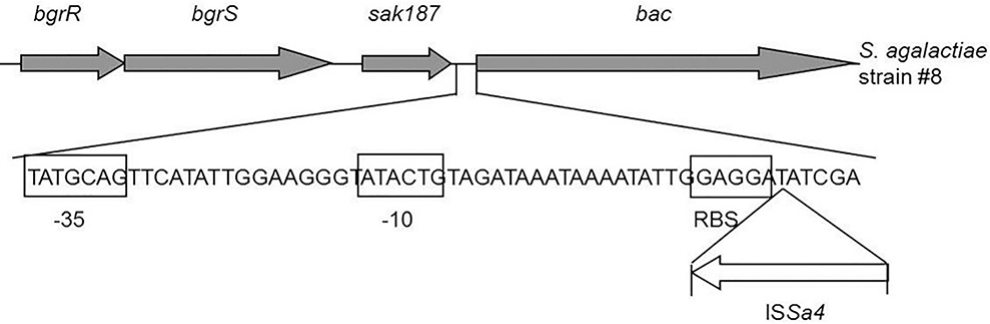
Schematic representation of the IS*Sa4* insertion in regulatory region of the *bac* gene in the strain #8. The sequences -35, -10, and ribosomal binding site are indicated in boxes.

PCR analysis of the *bac* gene in the strain #8 using the primers 0524–76 and L6 revealed an amplicon which was approximately 1 kb larger than expected size. Sequencing of this DNA fragment using the primers listed in the Table 1 identified an integration of IS*Sa4* upstream the *bac* gene that occurred just after the ribosomal binding site GAGGA (Fig 2). As deduced, integration of this insertion sequence in the regulatory region of *bac* gene resulted in inability to activate the *bac* gene transcription and repression of β antigen synthesis. The corresponding nucleotide sequence in the strain #8 has been deposited into the GenBank database under accession number KF444678.

Additionally, all 48 *bac* gene positive strains that express β antigen were tested for the presence of IS*Sa4*. Using PCR and nucleotide sequencing, in different strains IS*Sa4* was found to be integrated in exonuclease gene, phage integrase gene, hypothetical protein genes, oxidoreductase gene, membrane protein gene, competence protein gene, among others, but not upstream the *bac* gene. Therefore, an integration of IS*Sa4* upstream *bac* gene in the strain #8 indeed is a reason for abrogation of β antigen expression.

### Point mutation in the *bac* gene of the strain #06/08

**Fig 3:**
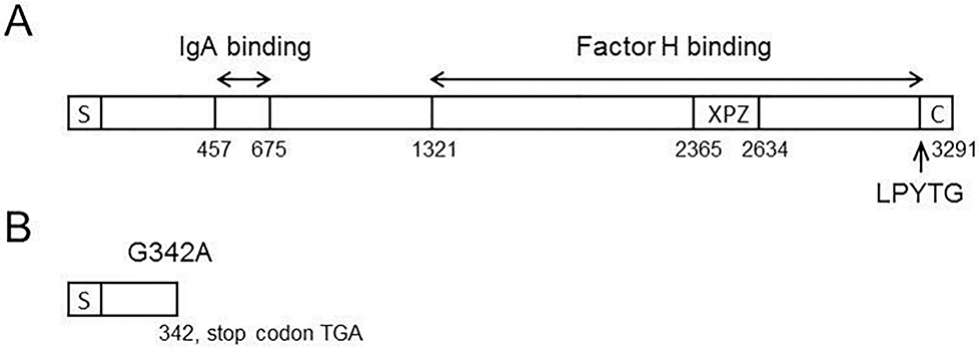
Schematic representation of the *bac* gene in the strain A909 that expresses β antigen (A), and the *bac* gene in the strain #06/08 that did not produce β antigen (B). The numbers indicate nucleotide positions. The signal sequence, C-terminal end, XPZ and LPYTG motifs, and IgA binding and factor H binding domains indicated according to [20].

As mentioned above, the strain #06/08 did not produce β antigen. In order to analyze the lack of β antigen expression in this strain, the entire *bac* gene including promoter region has been sequenced. Subsequent analysis of the *bac* gene in the strain #06/08 revealed the single point mutation G342A compared to the strains in which β antigen was expressed. This point mutation resulted in formation of stop codon TGA and therefore truncation of β antigen (Fig 3). The nucleotide sequence of the *bac* gene in the strain #06/08 has been deposited into the GenBank database under accession number KF444679.

### Frameshift of the *bgrR* gene in the strain A49V

**Fig 4:**
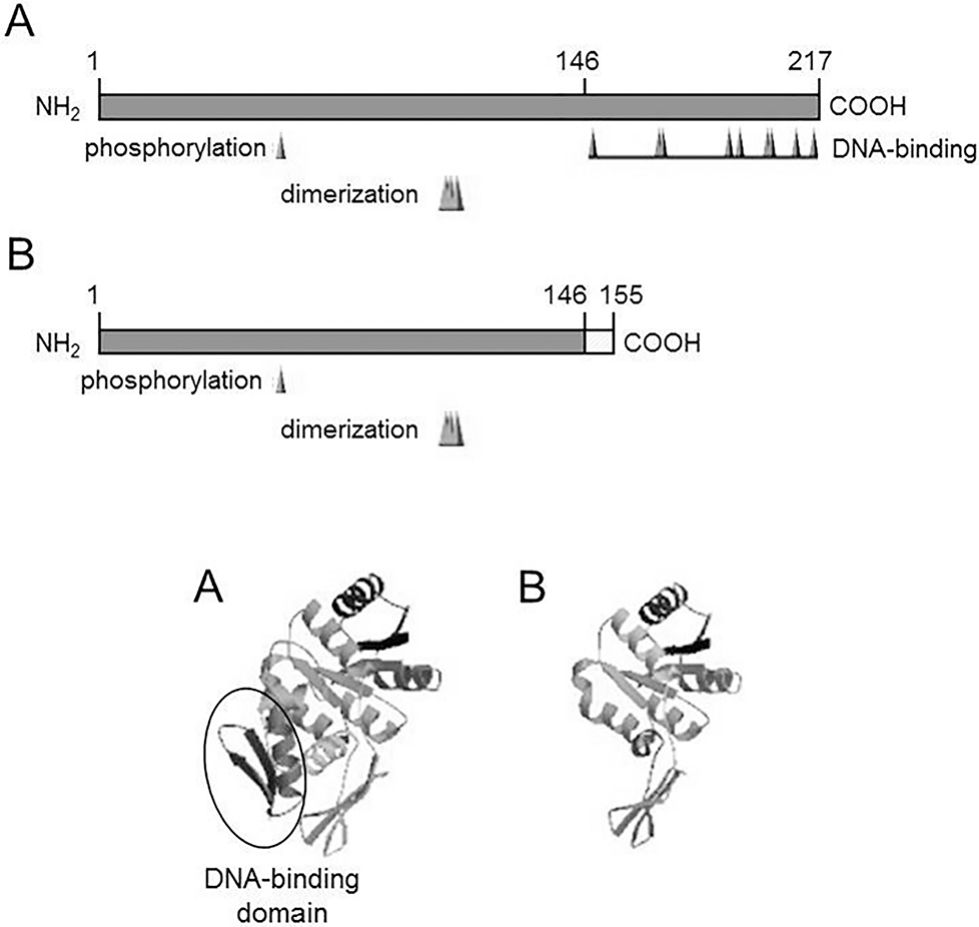
Schematic representation of the BgrR proteins and the corresponding three-dimensional structures in the strains 168/00 (A) and A49V (B). The numbers indicate aminoacid positions. Phosphorylation, dimerization and DNA-binding sites are shown.

PCR and sequencing analyses of the *bac* gene and its promoter region in the strain A49V did not reveal any differences compared with those in the strains expressing β antigen. In order to analyze if the lack of β antigen expression can be associated with mutation in the genes of two-component regulatory system BgrRS, the genes *bgrR* and *bgrS* were sequenced and compared with those in the strain 168/00 expressing β antigen. As result, analysis of the *bgrR* and *bgrS* gene sequences in A49V strain revealed single nucleotide (G) deletion in position 439 of *bgrR* compared to the *bgrR* gene in the strain 168/00 (GenBank accession number FJ890928). This deletion resulted in the frameshift of *bgrR* gene. As deduced, the changes resulted in the replacement of 72 amino acids by 10 other amino acids at the C-terminal end of BgR protein. Importantly, all 9 amino acids involved in DNA binding activity of BgrR protein as well as entire DNA-binding domain were lost in the strain A49V (Fig 4). Nucleotide sequence of the *bgrR* gene in A49V strain has been deposited into the GenBank database under accession number HQ840774.

### Point mutations in the *bgrR* and *bgrS* genes of the strains #122 and #128

Given that two-component regulatory system BgrRS is responsible for the *bac* gene expression, the sequences of the *bgrR* and *bgrS* genes of #122 and #128 strains were determined. Compared to the control strain 168/00, in both #122 and #128 strains *bgrR* gene contained single nucleotide substitution G560A, and *bgrS* gene contained two nucleotide substitutions C474T and G755A. As deduced, two of these mutations resulted in the replacement of amino acids in BgrR (Arg187Lys) and BgrS (Arg252Gln) proteins and could result in formation of the functionally inactive BgrR and/or BgrS proteins. Nucleotide sequences of *bgrR* and *bgrS* genes in both #122 and #128 strains have been deposited into the GenBank database under accession numbers KF472175 and KF472176, respectively.

### Complementation of the strains A49V, #122, and #128 by functionally active two-component regulatory system BgrRS

**Fig 5:**
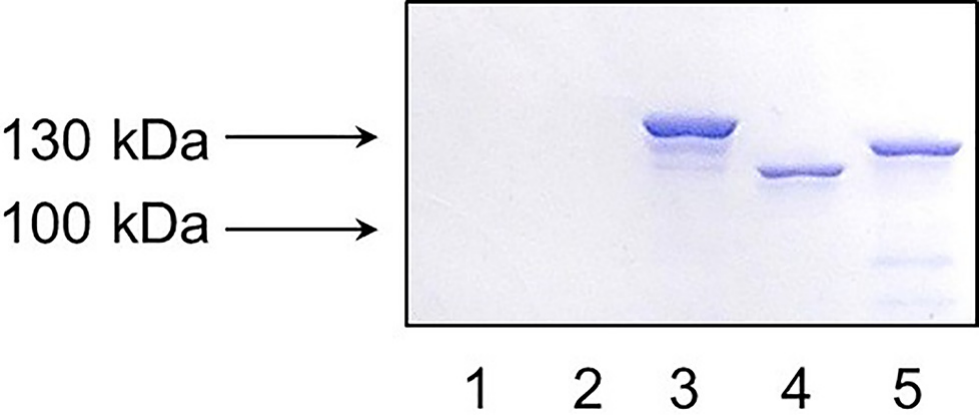
SDS-PAGE of *S*. *agalactiae* cell lysates followed by western-blotting with conjugated human IgA-horseradish peroxidase. Lane 1: strain A49V; lane 2: strain #122; lane 3: strain 168/00; lane 4: strain A49V/BgrRS; lane 5: strain #122/BgrRS.

In order to prove that abrogation of β antigen expression in the strains A49V, #122, and #128 were associated with mutations in the genes of BgrRS two-component system, the expression of functionally active alleles of both *bgrR* and *bgrS* genes were restored in the strains A49V, #122 and #128. To do so, the DNA fragment containing promoter region and the *bgrR* and *bgrS* genes of the control strain 168/00 were cloned into expression vector pAT29 as described in Materials and Methods. The resultant plasmid, pBgrRS(P), were used to transform each of the strains A49V, #122, and #128. The spectinomycin resistant clones were screened, the recombinant plasmids were isolated, and the corresponding complemented strains were named as A49V/BgrRS, #122/BgrRS, and #128/BgrRS. Construction of the strains was confirmed by PCR and nucleotide sequencing (data not shown). SDS-PAGE and western-blotting revealed restoration of β antigen in each of the complemented strains (Fig 5).

### Virulent properties of the strains A49V and A49V/BgrRS

**Fig 6:**
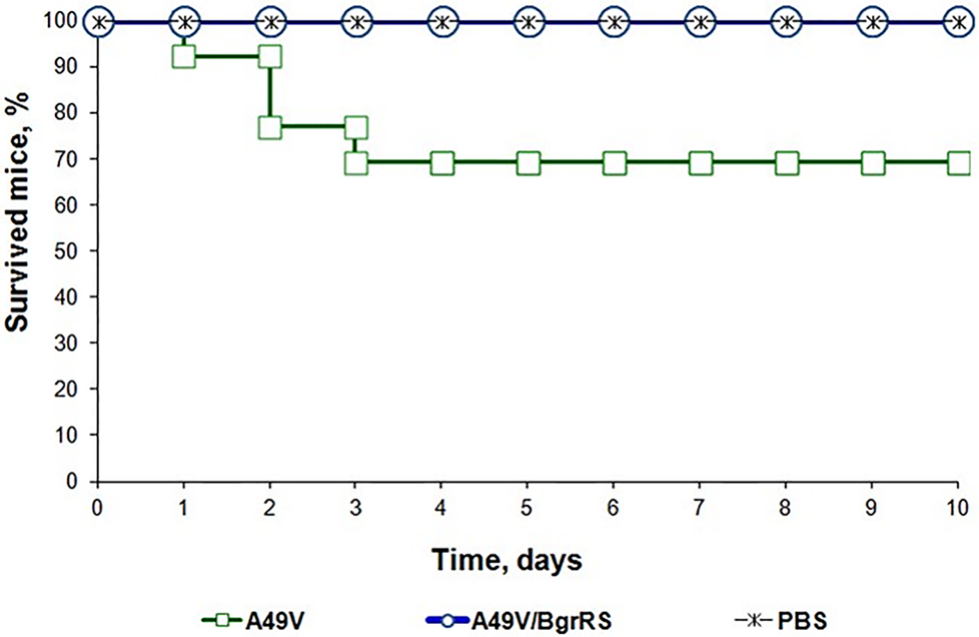
Mortality rates of laboratory mice due to *S*. *agalactiae* intra-peritoneal infection.

The virulent properties of the strain A49V with non-functional BgrRS, and the complemented strain A49V with functional BgrRS (Fig 5) were compared employing *in vivo* infection model as described in Materials and Methods. As result, infection of laboratory animals with the strain A49V resulted in the death of 4 out of 13 animals, while the strain A49V/BgrRS was found to be avirulent, P ≤ 0.05 (Fig 6). Together, these data indicate that BgrRS two-component system negatively controls virulent properties of *S*. *agalactiae*.

## Discussion

Expression of the virulent factors by pathogenic bacteria is extremely important for successful adaptation to human host, colonization of the tissues and organs, and fast infectious process [4]. Depending on the growth phase or the stage of interaction with the host, pathogenic bacteria activate or repress different virulence factors [16]. Potential absence of the virulence factor expression can be explained by numerous reasons. First, protein expression can be negatively regulated, in particular, by two-component systems. In addition, the deletions, insertions, duplications within the gene structure can result in the frame-shift and the changes in virulence factor sequence, conformation, and functional activity. Finally, an increase or decrease in protein expression can reflect abnormal transcriptional regulation.

The β antigen, which is considered as a virulent factor of *S*. *agalactiae*, is encoded by the *bac* gene and expressed during all the phases of growth [36]. However, 5 out of 140 *S*. *agalactiae* strains under present study did not express β antigen. In 2 of these 5 strains the disruption of the β antigen expression was associated with sequence of the *bac* gene. In the strain #06/08 the point mutation in the *bac* gene resulted in formation of stop codon TGA. Computer analysis identified that truncated form of the β antigen in this strain can consists of 351 aminoacids. As deduced, this truncated form does not possess IgA binding domain and, therefore, it can not be detected with SDS-PAGE followed by western-blotting. Previously, truncated non-functional forms of β antigen due to the mutations in the *bac* gene were also described [37, 38].

In the strain #8 an absence of β antigen expression was associated with insertion of IS*Sa4* in the regulatory region of *bac*. Interestingly, the virulence genes or DNA regions associated with the control of virulent properties of *S*. *agalactiae* are frequent targets for insertion sequences such as IS*1548*, IS*Sa4* and IS*1381* [39–43]. In particular, one of the IS*1381* copies was found to be integrated in the *bac* gene [44]. The question why insertion of ISs often happens in the virulence regions requires further analysis.

Identification of the molecular basis of virulence gene regulation can be very useful not only for understanding of pathogenesis of bacterial and viral diseases, but also for the target drug design and application of antibacterial and antiviral drugs that selectively block expression of virulence factors. As determined, three *bac* gene positive strains without expression of β antigen (A49V, #122, #128) carried out functionally active *bac* gene alleles, but were characterized by the mutations in *bgrR* and *bgrS* genes. According to the BLASTp analysis (http://blast.ncbi.nlm.nih.gov/Blast.cgi), in the strain A49V mutations in the *bgrR* gene resulted in the loss of all 9 amino acids involved in DNA binding activity of BgrR protein, while in the strains #122, #128 the arginine at position 187 was replaced with lysine. The arginine at position 187 is involved in formation of DNA binding domain, and therefore it seems to be crucial for regulatory activity of BgrR. In the strains #122 and #128 mutation in *bgrS* gene resulted in mutation Arg252Gln, and therefore, this amino acid can be potentially involved in functional activity of BgrS.

Complementation of the strains A49V, #122, and #128 with *bgrR* and *bgrS* genes restored expression of the *bac* gene and indicated that BgrRS two-component system is extremely important for *S*. *agalactiae*.

Intraperitoneal infection of laboratory mice demonstrated difference in the virulent properties of the strain A49V/BgrRS in which BgrRS two-component system and β antigen expression was restored compared to the strain A49V. It is not surprised because usually inactivation of TCSs in *S*. *agalactiae* affects virulence. However, in the previous studies the strains, in which TCS (CovS/CovR, DltR/DltS, RgfA/RgfC) genes were inactivated, demonstrated attenuated virulence [7–11]. Therefore, these TCSs positively control virulence. In our study attenuated virulence was observed when the functionally active *bgrR* gene allele was restored. Given that one of the functional roles of BgrRS two-component systems is a control of *bac* gene transcription (β antigen expression) [14] these data give a novel clue for understanding of the role of BgrRS TCS and possibly β antigen in adaptation of *S*. *agalactiae* to the human host and virulence. Previously when we inactivated two-component system genes *bgrR* and *bgrS* in the strain 168/00 we also observed that the strain with functionally active BgrRS system was characterized by decreased virulence [14]. It is quite possible that acquisition of the *bac*, *bgrR* and *bgrS* genes within the virulence island [12] did not provide additional virulent properties for the “struggle” with human host and causing severe forms of diseases, but, on the contrary, it provided an ability for continuous asymptomatic colonization of the human. Given, that two-component systems usually affect transcription of the numerous genes [4], it is expected that *bac* gene not only target for BgrRS. Identification of all the BgrRS TCS affected genes, for example, with microarrays, can help to understand the mechanisms of adaptation of *S*. *agalactiae* in its natural ecological niches such as vaginal epithelium or prostatic liquid of human and milk of the dairy cows. This adaptation and mimicry can explain the quick spreading of the *bac* gene positive strains in human population.

In conclusion, the data presented here demonstrate that the disruption of β antigen expression in the strains under study are associated with: *i*) insertion of IS*Sa4* upstream the *bac* gene just after the ribosomal binding site; *ii*) point mutation G342A resulting a stop codon TGA within the *bac* gene and a truncated form of β antigen; *iii*) single deletion (G) in position 439 of the *bgrR* gene resulting in a frameshift and the loss of DNA-binding domain of the BgrR protein, and potentially *iv*) point mutations causing single amino acid substitutions in BgrR (Arg187Lys) and BgrS (Arg252Gln) proteins. In addition, the results indicate that BgrRS two-component system negatively controls virulent properties of *S*. *agalactiae* and give a novel clue for understanding of *S*. *agalactiae* adaptation to the human host.

## Supporting Information

S1 Table Arrive checklist “Natural mutations in *Streptococcus agalactiae* resulting in abrogation of β antigen production.” (DOCX)
