# Supplementary material for: Natural Mutations in Streptococcus agalactiae Resulting in Abrogation of β Antigen Production
Source: PLoS One. 2015 Jun 5;10(6):e0128426. doi: 10.1371/journal.pone.0128426 (PMC4457541; doi:10.1371/journal.pone.0128426)
Supplement: S1 Table — (DOCX) [file pone.0128426.s001.docx]

|  | ITEM | RECOMMENDATION | Section/  Paragraph |
| --- | --- | --- | --- |
| Title | 1 | Natural mutations in *Streptococcus agalactiae* resulting in abrogation of β antigen production | Title |
| Abstract | 2 | *Streptococcus agalactiae* genome encodes 21 two-component systems (TCS) and a variety of regulatory proteins in order to control gene expression. One of the TCS, BgrRS, comprising the BgrR DNA-binding regulatory protein and BgrS sensor histidine kinase, was discovered within a putative virulence island. BgrRS influences cell metabolism and positively control the expression of *bac* gene, coding for β-antigen at transcriptional level. Inactivation of *bgrR* abrogated *bac* gene expression and increased virulence properties of *S. agalactiae*. In this study, a total of 140 strains were screened for the presence of *bac* gene, and the TCS *bgrR* and *bgrS* genes. A total of 53 strains carry the *bac, bgrR* and *bgrS* genes. Most of them (48 strains) expressed β-antigen, while five strains did not express β-antigen. Three strains, in which *bac* gene sequence was intact, while *bgrR* and/or *bgrS* genes had mutations, and expression of β-antigen was absent, were complemented with a constructed plasmid pBgrRS(P) encoding functionally active *bgrR* and *bgrS* gene alleles. This procedure restored expression of β-antigen indicating the crucial regulatory role of TCS BgrRS. The complemented strain A49V/BgrRS demonstrated attenuated virulence in intraperitoneal mice model of *S. agalactiae* infection compared to parental strain A49V. In conclusion we showed that disruption of β antigen expression is associated with: *i*) insertion of IS*Sa4* upstream the *bac* gene just after the ribosomal binding site; *ii*) point mutation G342A resulting a stop codon TGA within the *bac* gene and a truncated form of β antigen; *iii*) single deletion (G) in position 439 of the *bgrR* gene resulting in a frameshift and the loss of DNA-binding domain of the BgrR protein, and *iv*) single base substitutions in *bgrR* and *bgrS* genes causing single amino acid substitutions in BgrR (Arg187Lys) and BgrS (Arg252Gln). The fact that BgrRS negatively controls virulent properties of *S. agalactiae* gives a novel clue for understanding of *S. agalactiae* adaptation to the human. | Abstract |
| INTRODUCTION | | | |
| Background | 3 | Expression of *S. agalactiae* virulence factors is controlled at transcriptional level by two-component regulatory systems (TCSs) and global transcriptional regulators. BgrRS TCS of *S. agalactiae* was found to be located within the putative virulence island possibly acquired recently by *S. agalactiae* providing certain selective advantages to *S. agalactiae*. The BgrRS TCS co-transcribed genes *bgrR* and *bgrS* are adjacent to the virulence gene *bac* encoding for the surface β antigen. This surface protein has capacity to bind IgA and factor H of complement, and is thought to be an important virulence factor. However, similarly to other virulence factors of pathogenic bacteria, the expression of β antigen may (or may not) be variable in different strains or even be abrogated. To test this hypothesis, a collection of *S. agalactiae* strains was analyzed in this study in order to reveal the strains without β antigen expression, to discover the reasons of abrogated β antigen expression, and to further study functional role of BgrRS TCS and β antigen in *S. agalactiae* virulence. | Introduction |
| Objectives | 4 | The purposes of this study were to analyze an epidemiological diverse collection of GBS to select for strains without β antigen expression, to subsequently reveal the causes for the abolished β antigen expression, and to further study the functional role of BgrRS TCS and β antigen in *S. agalactiae* virulence by using an *in vivo* streptococcal infection model. | Introduction |
| METHODS | | | |
| Ethical statement | 5 | All the experiments were performed according to the Protocol No. 3 (2011) approved by Animal Care Unit Committee, Institute of Experimental Medicine, Russia. The animals were sacrificed by CO_2_ asphyxiation and cervical dislocation, and all the efforts were done to minimize suffering. | Materials and methods |
| Study design | 6 | Two different *S. agalactiae* suspensions (of one clinical strain with a frameshift mutation of *bgrR* gene and of the complemented strain with functionally active two-component regulatory system BgrRS) in the volume of 0.5 ml were injected intra-peritoneally into anesthetized (inhalation of isoflurane) mice of the experimental groups. As control, 0.5 ml of PBS was injected into a control group of animals. | Materials and methods,  Results |
| Experimental procedures | 7 | Overnight cultures of *S. agalactiae* were harvested and suspended at a concentration of 1 to 5 x 10^8^ CFUs in 0.5 ml of PBS and used for intra-peritoneal injection to experimental animals. Observation was done for 10 days, and the animals were monitored four times a day during the period 9:00 am – 18:00 pm. The endpoints for sacrifice were defined as follows: extreme presentation of the clinical signs of infection four times a day (huddling, hunched posture, ruffled fur, tachypnea); severe hypothermia as indicated by a temperature of 34°C (~4.5°C below normal) in the days following bacterial challenge; weight loss equal to 25% of starting weight; and/or severe illness predictive of death or the moribund state. The mice demonstrating extreme signs of illness were immediately humanely euthanized by CO_2_ asphyxiation and cervical dislocation and considered to have succumbed to the infection within 24 hours of achieving aforementioned endpoints. These endpoint criteria have been incorporated to avoid the use of death as endpoint. At the end of experiments, all the remaining mice were also sacrificed. In order to confirm the death of the animals from *S. agalactiae* infection, the spleens of died or sacrificed animals were isolated and homogenized in PBS. The large number of bacterial CFUs was isolated from the spleens of died animals. They were found to be group B streptococcal gram-positive, catalase negative, and identified as *S. agalactiae*. | Materials and methods,  Results |
| Experimental animals | 8 | Outbred ten-week old (14-16 g) healthy male mice (Rappolovo Animal Facility, Russia) were used in all the experiments. | Materials and methods |
| Housing and husbandry | 9 | The animals were housed according to standard animal laboratory conditions. The animals were housed in polycarbonate cages. Free access to balanced food and water was provided. | Materials and methods |
| Sample size | 10 | Each experimental group of animals contained 13 mice. The control group contained 10 mice for the control study. All the experiments were done in triplicates. The mortality rates of laboratory mice due to *S. agalactiae* intra-peritoneal infection were evaluated and representative data are shown in the Figure 6. | Materials and methods |
| Allocating animals to experimental groups | 11 | In each experiment two groups of the animals were used, i.e., experimental and control. All the animals had the similar weight and health conditions, and they were randomly distributed between experimental and control groups. | Materials and methods |
| Experimental outcomes | 12 | The experimental outcomes of the study were either survival animals or the reaching animals the death endpoints. | Materials and methods,  Results |
| Statistical methods | 13 | Statistical analysis included Kaplan-Meier survival curve and the log-rank test. | Materials and methods |
| RESULTS | | | |
| Baseline data | 14 | Ten-week old (14-16 g) healthy male mice were used. Inhalation of isoflurane (2.5%) was used for anesthesia prior to inoculation with bacteria. | Materials and methods |
| Numbers analysed | 15 | Together, 26 mice were used for the experimental study and 10 mice were used in the control study. | Materials and methods |
| Outcomes and estimation | 16 | All the data were statistically significant (P value less than 0.05). | Results |
| Adverse events | 17 | In order to avoid adverse events: i) all the animals were maintained in the quarantine for two weeks prior the experiment, and ii) conditions of the animals were monitored four times a day during the period 9:00 am – 18:00 pm. | Materials and methods |
| DISCUSSION | | | |
| Interpretation/scientific implications | 18 | A49V/BgrRS strain of *S. agalactiae* was avirulent in mice due to the presence of active *bgrR* and *bgrS* gene alleles. This strain could be used to protect mice challenged with virulent strains. The fact that BgrRS was shown to negatively control virulent properties of *S. agalactiae* gives a novel clue for understanding of *S. agalactiae* adaptation to the human. | Discussion |
| Generalisability/translation | 19 | The study design can be used for analysis of the functional role of any bacterial genes in the expression of virulent phenotype. |  |
| Funding | 20 | The study was done using the budgets of the Institute of Experimental Medicine, Saint-Petersburg, Russia and of R&D projects funded by European Union (FP7- Specific programme 'People’) and Fundacão para a Ciência e Tecnologia, Portugal. The funders were not involved in the study in any way. |  |
